# Endodontic Treatment of Fused Teeth with Talon Cusp

**DOI:** 10.1155/2014/738185

**Published:** 2014-01-20

**Authors:** Shima Sadat Miri, Hakimeh Ghorbani, Anousheh Rashed Mohassel

**Affiliations:** ^1^Department of Endodontics, Faculty of Dentistry, Kermanshah University of Medical Sciences, Kermanshah 67147, Iran; ^2^Department of Oral and Maxillofacial Radiology, Faculty of Dentistry, Babol University of Medical Sciences, Babol 4717647745, Iran; ^3^Dental Research Center, Department of Pedodontics, Faculty of Dentistry, Birjand University of Medical Sciences, Birjand 97178, Iran

## Abstract

Tooth anomalies are rare phenomena that may be reported by patients as chief complaints or may be discovered by dentists in the oral examination. In a few cases, rare anomalies are found together in one mouth. Decision to treat such anomalies depends on whether or not they interfere with function and esthetics and also the patient's preference. In the present case, a 19-year-old male presented with two right maxillary fused central and lateral incisors and a geminated left maxillary central incisor. A talon-like projection was found at the junction site of the fused teeth. His chief complaint was sensitivity to cold at the site of the giant fused tooth. This report describes the process of diagnosis and treatment of the two anomalies according to patient preference and needs.

## 1. Introduction

Clinical tooth anomalies are deviations in normal appearance that may involve color, shape, size, or number of teeth and may be found in oral examination by a dentist [1]. In your clinical examinations, rarely, you may face a giant tooth with a large mesiodistal width. Commonly, this phenomenon is referred to as “double teeth.” In definition “double teeth” may occur as either gemination or fusion.

Gemination and fusion are developmental anomalies with the prevalence rate of 0.1% and 0.5% in permanent and primary dentition, respectively. Gemination happens as a result of an unsuccessful attempt of tooth germ to divide which usually leads to a single root, root canal, and a large bifid crown with a common pulp chamber. Fusion is the union of hard tissue between two developing teeth. Fused teeth may share a common pulp chamber or have separate root canals and chambers. Fusion may occur between two normal adjacent teeth or a normal and a supernumerary tooth. In the latter case, differentiation between gemination and fusion is difficult, as a strong tool of differentiation (tooth count) is missed [2].

Another tooth anomaly in shape is called “talon cusp.” Talon cusp appears as a hard tissue projection usually rising from cementoenamel junction on the lingual surface and extending at least half the way to the incisal edge of anterior teeth. This projection may contain normal enamel, dentin, and varying amounts of pulp tissue. It mostly involves permanent maxillary lateral incisor with equal prevalence in both sexes. A wide range of less than 1% to 7.7% prevalence has been reported in different populations [3, 4].

Gemination, fusion, and talon cusp are rare developmental anomalies with unknown etiologies. Both genetic and environmental, local and systemic factors are assumed to be responsible. Talon cups may occur because of an outfolded dental lamina during morphodifferentiation stage. Genetic influence is potentiated by occurrence in close family members [3]. Many theories have been proposed about the etiology of double teeth. Metabolic, inflammatory, and even traumatic etiologies have been proposed. A relationship between twinning and odontoma has been suggested [5]. When rare anomalies come together, they become even rarer. As the exact etiology of irregularities in tooth development which lead to dental anomalies remains unclear yet, their simultaneous occurrence may be at the same time predicted, and a guideline to create new or reject previous theories. Besides, although mostly asymptomatic, anomalies in shape and size may cause functional and esthetic problems or periodontal and pulp tissue involvement which requires a multidisciplinary treatment [2].

This report presents a rare case of fusion between right maxillary central and lateral incisor and a left geminated maxillary central incisor with a talon cusp with pulp involvement.

## 2. Case Description

**Figure 1:**
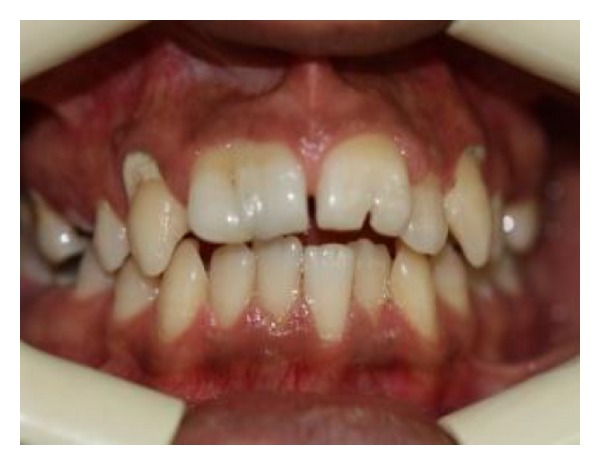
Intraoral facial view.

**Figure 2:**
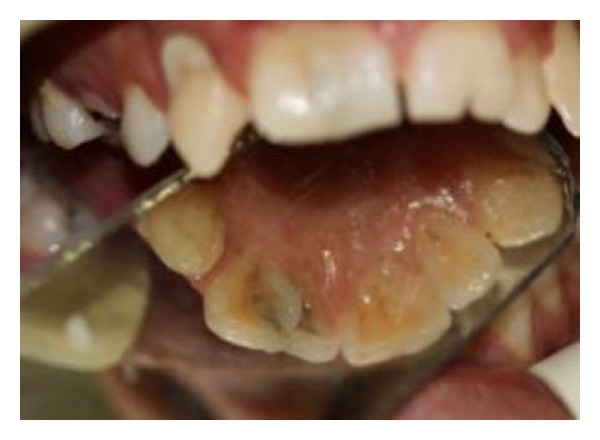
Intraoral palatal view.

**Figure 3:**
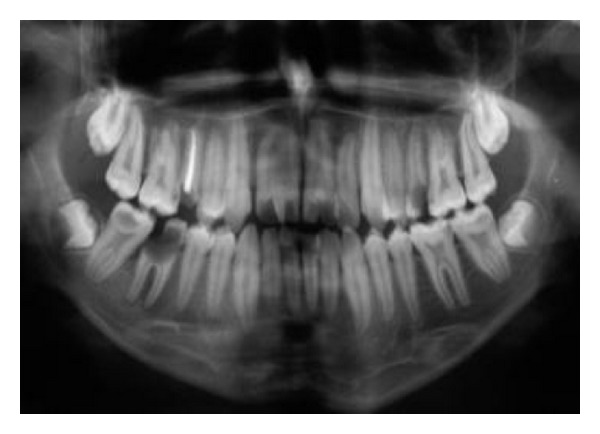
Panoramic view.

A 19-year-old male was referred to the Endodontic Department of Babol Dental College with the chief complaint of dental pain and sensitivity to cold in the anterior maxillary region. The patient appeared physically healthy, but mild mental retardation was obvious. In the medical history, he had childhood asthma which ameliorated by the age of seven. No history of trauma or hereditary involvement was reported. The patient showed a straight profile and a symmetric face without abnormal findings in extraoral examinations. Clinical intraoral examinations revealed permanent dentition with poor oral hygiene and multiple caries. The molar relationship was Angle class I in both sides. Maxillary and mandibular arcs were U-shaped with both midlines on (Figure 1). In the anterior maxillary region, we came to the rare finding of twinning in both central incisors. Both centrals showed an abnormally large mesiodistal width. No lateral incisor existed in the right maxillary region. Instead, a mesiodistally large central existed with a dental projection on its palatal side extending from the cingulum and ending at a small groove in the incisor edge (Figures 1 and 2). At the left side of the anterior maxilla, both central and lateral incisors existed. The left maxillary incisor also showed a large mesiodistal width with a large groove on the palatal surface that extended up to the incisal edge (Figures 1 and 2). There was no tooth mobility and no tenderness to percussion and palpation tests. A severe response to cold and electric tests was observed in the anterior maxillary incisor teeth. History taking revealed that he has had a previous endodontic treatment of a maxillary premolar in the same department. Reference to his file showed that his mother had been advised to refer to restoring caries in other teeth. She had also been suggested to seal the palatal grooves of the left incisor and esthetic treatment in maxillary anterior region. Because of poor socioeconomic status, further treatment was delayed until pain occurred. A panoramic radiograph was prescribed to detect possible further anomalies and define the condition in the anterior maxillary region (Figure 3). In the right maxillary, there were two crowns fused by dentin with one root and two canals. One tooth was missing in the right maxillary quadrant. In the left maxillary region, there was one bifid crown with one root and one root canal. No further anomaly was observed. No tooth was missing in the left maxillary quadrant. According to clinical and radiographic findings and tooth count in maxillary quadrants, dental fusion between right maxillary central and lateral incisor with a talon cusp and gemination in left maxillary central incisor were diagnosed.

According to clinical and radiographic findings, caries initiating from the grooves around the talon cusp reached the pulp chamber, and irreversible pulpitis was diagnosed in the right maxillary fused incisors. Endodontic treatment of right incisors accompanied by composite esthetic restoration in both maxillary sides was suggested to the patient and his mother to alleviate pain and make a better appearance. As the patient preferred, our treatment was limited to endodontic treatment and restoration of painful teeth, right maxillary fused central and lateral incisors.

**Figure 4:**
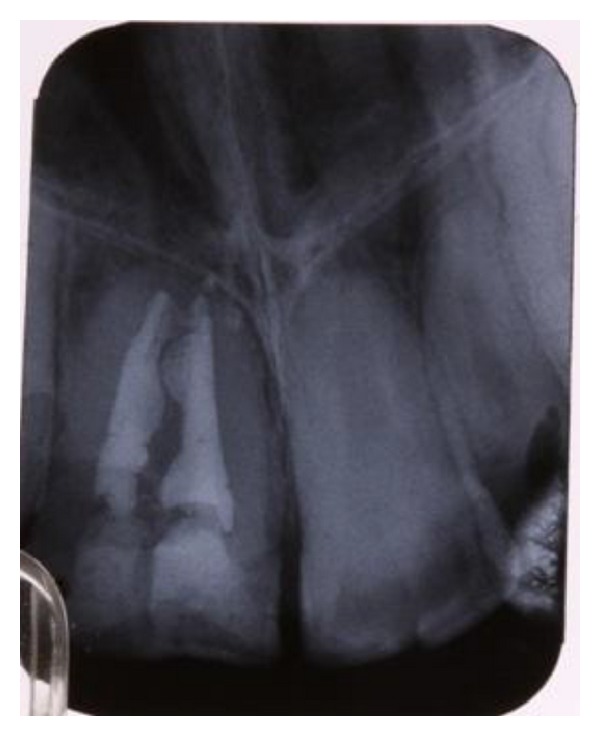
Periapical view of the treated canal.

**Figure 5:**
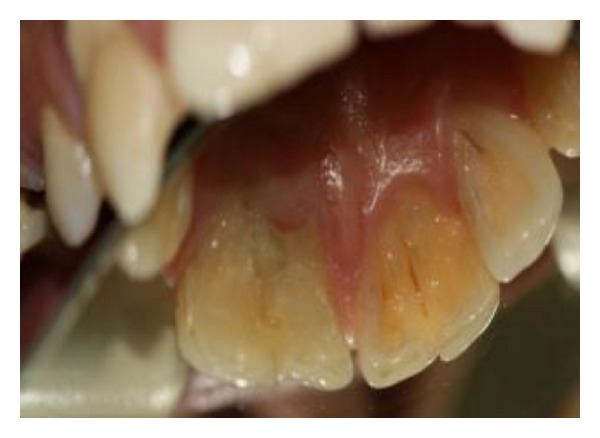
Restored palatal view.

Local anesthesia containing 1.8 mL lidocaine and 1/80000 epinephrine (Daroo-Pakhsh Co., Iran) was injected in the labial sulcus and a rubber dam (Hu-friedy, USA) was inserted. Two Access cavities were prepared by a fissure bur number 0.8 (Tees-Karvan, Iran). Pulp tissue was extirpated by a barbed broach (Dentsply Maillefer, Ballaigues, Switzerland). Working lengths of both canals were determined by K-File number 35 (Mani Inc., Japan) and a periapical radiograph. Canals were irrigated by 0.9% normal saline and sodium hypochlorite 2.5%. Cleaning and shaping was completed using a step back technique up to K-file number 80 and number 70 for the central and lateral incisor, respectively. As the patient was beginning to be uncomfortable and uncooperative, the canals were dried by sterile papers, calcium hydroxide was placed in root canals, the access cavities were temporized with cavit (Golchay Co., Iran), and the patient was dismissed. After one week, the patient returned for obturation. No pain or sensitivity during the week was reported. The canals were obturated by thermoplastic vertical condensation technique using gutta percha cones (Meta Biomed Co., Korea) and AH26 (Dentsply, USA) as a sealer (Figure 4). Access cavities were restored by universal light cure composite resin (3 M, ESPE) (Figure 5). The patient was asymptomatic in the six-month followup.

## 3. Discussion

Previous reports of gemination and fusion have mentioned chief complaints about unsightly appearance regarding the big ugly tooth or anterior crowding [2, 4, 6]. Other problems include occlusal interference, possibility of caries development in the grooves of the junction site, and eventual irreversible pulpitis [1–6]. Talon cusps are other anomalies that are less recognized by the patients as they mostly appear on the lingual surface of anterior teeth. Yet they also increase the risk of caries development in the grooves around the projection and further irreversible pulpitis. Occlusal interference is more probable when the talon cusp is located on the palatal surface of maxillary incisors. Occlusal interference may lead to improper displacement of teeth. Periodontal problems due to tooth movement have been reported. Facial talon cusps, especially on maxillary teeth, bring esthetic considerations [3, 7, 8]. Treatment of these anomalies is directly related to chief complaints and dental risks. The grooves may be sealed by fissure sealants or flowable composites. Episodic talon cusp reduction and fluoride therapy can solve mild occlusal interferences. In severe cases, a complete composite restoration may be required.

In the present case, the patient reported no concern about the appearance. There were no occlusal interferences or periodontal problems. He had been previously advised to seal the palatal grooves of twinned teeth. Unfortunately, the situation had been neglected and irreversible pulpitis occurred. Neither the patient nor his parents showed any interest in restoring the shape of teeth because of additional expenses. On preparing access cavities, the talon cusp was completely removed and after endodontic treatment the teeth were restored by composite resin.

Although the presence of two giant incisors instead of separated lateral and central incisors gives an unsightly appearance that dissatisfies most of the patients, our young patient suffering mild mental retardation did not have any concern about his appearance. As the patient's best is always a priority, we did not insist on esthetic reconstruction and sufficed to alleviate pain.

Regarding rare cases, previous reports with similarities provide valuable guidance. This, along with considering the patient concern and willing, determines our final treatment plan as in the present case.
